# Multifunctional self-emulsifying drug delivery system: an efficient strategy for oral delivery of therapeutic peptides and proteins

**DOI:** 10.1080/10717544.2025.2579137

**Published:** 2025-12-10

**Authors:** Guofei Li, Changyu Shao, Yuhao Zhang, Jinguo Li, Puxiu Wang, Tianyang Ren

**Affiliations:** a Department of Pharmacy, Shengjing Hospital of China Medical University, Shenyang, China; b Department of Pharmacy, the First Affiliated Hospital of China Medical University, Shenyang, China

**Keywords:** Oral delivery, therapeutic peptides and proteins, lipidization, self-emulsifying drug delivery system

## Abstract

Oral delivery is the most preferred route for accessing bioactive peptides and proteins for disease treatment from the patient’s viewpoint. However, this is a great challenge because orally administered peptides and proteins are susceptible to the harsh gastrointestinal (GI) environment, various metabolic enzymes, and thiol/disulfide exchange reactions, and they cannot permeate the intestinal mucus and epithelial barriers. The self-emulsifying drug delivery system (SEDDS) has recently gained prominence because of its potential in the oral delivery of peptides and proteins. Stable payloads of hydrophilic proteins can be achieved in SEDDSs using feasible lipidization techniques, especially hydrophobic ion pairing. Upon entrapment in oily droplets derived from SEDDSs, protein drugs can be protected against enzymatic degradation and thiol/disulfide exchange reactions in the GI tract. After optimizing functional excipients and developing an efficient combination strategy, SEDDS droplets exhibit high mucus and membrane permeability and further enhance drug absorption and transport. The desired oral bioavailability and therapeutic effects of peptide and protein drugs could be achieved *in vivo*. This review presents the progress in the development of multifunctional SEDDSs for oral peptide and protein delivery. However, with the introduction of more novel multifunctional auxiliary agents and complicated structures to new-generation SEDDSs, more work is needed to identify the effects of excipients in the optimized combination and increase our knowledge of the fate of the excipients and the nanocarrier during absorption and transport. This knowledge will facilitate the future development of multifunctional SEDDSs for the oral application of therapeutic peptides and proteins.

## 1. Introduction

Since the discovery of insulin in the 1920s, peptide and protein drugs have attracted increasing attention as alternatives to conventional small-molecule drugs, and great developments have been made in preclinical research and clinical use. As bioactive macromolecular drugs, protein and peptide drugs are highly efficient, highly selective, have few adverse effects, and have good tolerability compared with small-molecule drugs. Despite these innate therapeutic advantages, it is widely acknowledged that their potential clinical uses have been partly hampered by their limited parenteral routes of administration. To date, therapeutic peptides and proteins available on the market are primarily administered *via* injection, which leads to inconvenience, poor patient compliance, and various risks. Among the various noninvasive routes of administration, the oral route is most preferred because of its high patient compliance, safety, and convenience. Therefore, pharmaceutical scientists have intensively searched for new drug delivery systems enabling the oral delivery of therapeutic peptides and proteins in recent decades. However, this is a great challenge because orally administered peptides and proteins are susceptible to acidic pH, gastrointestinal (GI) peptidases, and thiol/disulfide exchange reactions with endogenous thiols, and they rarely permeate the intestinal mucosa, primarily because of their large molecular sizes (Goldberg and Gomez-Orellana 2003; Moroz et al. 2016).

Self-emulsifying drug delivery systems (SEDDSs) are defined as isotropic mixtures of synthetic or natural oils, surfactants, solvents, and cosolvents/surfactants that form fine oil-in-water emulsions upon contact with the GI aqueous medium with slight peristaltic movement. As traditional lipid-based nanocarriers, SEDDSs improve the solubility and oral absorption of poorly water-soluble drugs. More importantly, SEDDSs have gained prominence in recent decades because of their potential in the oral delivery of hydrophilic peptides and proteins. Feasible lipidization techniques such as hydrophobic ion pairing (HIP), H-bond pairing, and prodrug formulations have been widely developed to increase the lipophilic character of these hydrophilic macromolecular therapeutics sufficiently, and these formulations have acceptable payloads in SEDDSs. Novel multifunctional auxiliary agents, such as muco-inert agents, charge-changing agents, cell-penetrating peptides, zwitterions, and their appropriate combinations, can overcome the limitations associated with SEDDSs in oral peptide and protein delivery. SEDDS technology can be used to achieve good payloads and protect against the harsh GI environment, improve intestinal mucus and cell permeation, and increase intestinal transport and absorption, leading to obviously increased oral bioavailability and therapeutic effects. The purpose of this review is to describe feasible lipidization techniques, opportunities for the oral delivery of peptide and protein drugs provided by novel SEDDSs, and their future potential.

## 2. Main barriers to the oral delivery of therapeutic peptides and proteins

### 2.1. Biochemical barriers in the GI tract (Moroz et al. 2016)

The GI tract is functionally optimized to digest food, absorb nutrients, and defend against exogenous pathogens, and it is an extremely complex environment in terms of physiological structures and chemical constituents. The biochemical barriers encountered in the oral delivery of therapeutic peptides and proteins primarily include acidic pH, various enzymes, surfactants, and exogenous or endogenous thiols.

The luminal pH in the GI tract varies greatly from the stomach to the terminal small intestine. Normally, the pH of gastric fluid ranges from 1 to 2, and the pH sharply changes from strongly acidic in the stomach to nearly neutral (pH 6–6.5) in the duodenum before increasing to 7–8 in the terminal ileum (Koziolek et al. 2016). Generally, most proteins are stable in a narrow pH range close to their isoelectric point. Therapeutic peptides and proteins must resist the highly acidic environment in the stomach before absorption, which mostly occurs in the small intestine. However, extremely low pH is known to degrade or decrease the activity of peptide and protein drugs by causing oxidation, deamidation, or hydrolysis (Cao et al. 2021). Moreover, these acidic conditions result in protonation and conformational alterations attributable to the increase in electrostatic repulsion, which further facilitates protein degradation by GI enzymes (Fuhrmann and Leroux 2014). In addition, metabolic enzymes, such as pepsin, most strongly degrade proteins at pH 2–3, but they are completely inactive at pH exceeding 5.

Various enzymes in the GI tract represent another obstacle to the oral delivery of peptide and protein drugs because most peptides and proteins are vulnerable to proteolytic degradation by digestive or mucosal enzymes (Goldberg and Gomez-Orellana 2003). In the stomach, enzymatic degradation is induced by pepsin, which is highly active in acidic environments. Pepsin tends to degrade proteins into small fragments by hydrolyzing peptide bonds at cleavage sites such as leucine and phenylalanine, and this degradation is facilitated by protein unfolding at extremely low pH values (Fuhrmann and Leroux 2014). Only a few peptide drugs, such as semaglutide and salmon calcitonin (sCT), have been verified to be absorbed from the stomach, although their gastric absorption is obviously less than that in the intestine. Intestinal enzymes, such as trypsin, chymotrypsin, elastase, carboxypeptidase A, and carboxypeptidase B, can induce rapid enzymatic cleavage of a majority of peptide drugs. Trypsin and chymotrypsin have the propensity to cleave peptide bonds at arginine, lysine, and aromatic amino acids (Fuhrmann and Leroux 2014). In addition, brush border membrane-bound enzymes containing aminopeptidase *N* and endopeptidase 24.11 and cytosolic enzymes of the GI epithelium are involved in the proteolytic degradation of peptide and protein drugs in the GI tract (Woodley 1994; Bernkop-Schnürch 1998).

Peptide and protein drugs generally contain thiol or disulfide substructures, which are highly susceptible to various thiols in the GI tract. Thiol/disulfide exchange reactions easily lead to the denaturation of therapeutic peptides and proteins. In addition to endogenous thiols, including the glutathione, homocysteine, cysteine, and cysteine subdomains of mucins in mucus, exogenous thiols from dietary food also constitute a dominant part of the GI sulfhydryl barrier. Thiols in fruits (4–136 nM/g) and vegetables (3–349 nM/g) include glutathione, cysteine, homocysteine, *N*-acetyl cysteine, and *γ*-glutamyl cysteine. The degradation of lanreotide by thiol/disulfide exchange reactions was studied in the presence of glutathione- or thiol-enriched casein peptones as dietary proteins (Ijaz et al. 2016). The results revealed that <10% of lanreotide remained after 1 h, and complete degradation of lanreotide was observed after 2 h. As reported in another study, desmopressin was also rapidly degraded in the GI tract *via* thiol/disulfide exchange reactions with glutathione (Schmitz et al. 2006). Moreover, peptide and protein drugs are potentially subject to denaturation by intestinal surfactants, such as bile salts.

### 2.2. GI mucus and epithelial barriers

GI mucus is a highly viscoelastic and adhesive gel layer covering the GI epithelium that efficiently protects exposed epithelial surfaces against pathogens and xenobiotics. The protective effect of the mucus layer in the GI tract is primarily attributed to its unique chemical and physical characteristics and continuous turnover. Mucus is a complex hydrogel composed of proteins, carbohydrates, lipids, salts, and other components, and its three-dimensional network structure formed by mucins as the main protein component naturally impedes the penetration of foreign macromolecules or particulates (Ensign et al. 2012). Although not all peptide and protein drugs are blocked by mucin networks with mesh sizes ranging from 10 to 200 nm, the high viscosity of mucus further decreases their diffusion through the mucus layer. Strong interactions between mucins and peptides might be generated *via* electrostatic force because of the highly negative charge of mucins, hydrogen bonding, hydrophobic interactions, and even van der Waals forces, which both hamper the mobility of large peptides in mucus and lead to their structural modification (Capaldo et al. 2017; Qi et al. 2018). Moreover, the mucus layer is continuous, being thickest in the colon (approximately 830 μm) and thinnest in the jejunum (approximately 123 μm) (Atuma et al. 2001). Mucus normally contains a firmly adherent viscous layer directly attached to the epithelium and a loosely adherent, less viscous layer distal to the epithelium. Constant turnover of the mucus layer serves to rapidly remove trapped peptide and protein drugs if they do not efficiently reach the deep mucus layer (Atuma et al. 2001; Ensign et al., 2012). Thus, the infiltration of peptide and protein drugs through the mucus layer is limited.

The GI epithelium beneath the mucus layer is considered the most challenging barrier for oral peptide and protein delivery. This continuous cell monolayer is composed of various functional cells, such as absorptive enterocytes, mucus-secreting goblet cells, enzyme-secreting Paneth cells, and microfold cells (M cells), for antigen and pathogenic particle transport. The tight junctions formed by multiprotein junctional complexes between two neighboring epithelial cells make the GI epithelium seamless and impermeable. Generally, peptides and proteins can be absorbed across this simple epithelium from the GI lumen through the transcellular route, paracellular route, and carrier-mediated transport. In fact, the transcellular permeation of peptides and proteins is extremely low because of their large molecular size and low lipophilicity. As previously reported, the membrane permeability of drugs decreases sharply when their molecular mass exceeds 500 Da, and the highly hydrophilic nature of peptides and proteins prevents them from partitioning into epithelial cell membranes (Goldberg and Gomez-Orellana 2003). Compared with normal epithelial cells, peptide and protein drugs are prone to be transported by M cells located in the follicle-associated epithelium *via* pinocytosis and phagocytosis. However, this transport is extremely limited, primarily because of the severely low distribution of M cells in the intestine. In fact, gut-associated lymphoid tissue (GALT) accounts for less than 10% of the total intestinal surface area, and only 10% of the epithelial cells in GALT are M cells (Trevaskis et al. 2015). Therefore, it is difficult for peptide and protein drugs to be absorbed *via* the transcellular route. Moreover, the paracellular route might be the preferred transport route, but paracellular spaces constitute only approximately 1% of the epithelial surface (Antosova et al. 2009). In addition, the water-filled pores of tight junctions for nutrient transport are sufficiently small to block the passage of drug molecules larger than 500 Da even in the fully open state.

## 3. Feasible techniques for loading therapeutic peptides and proteins in SEDDSs

**Figure 1.**
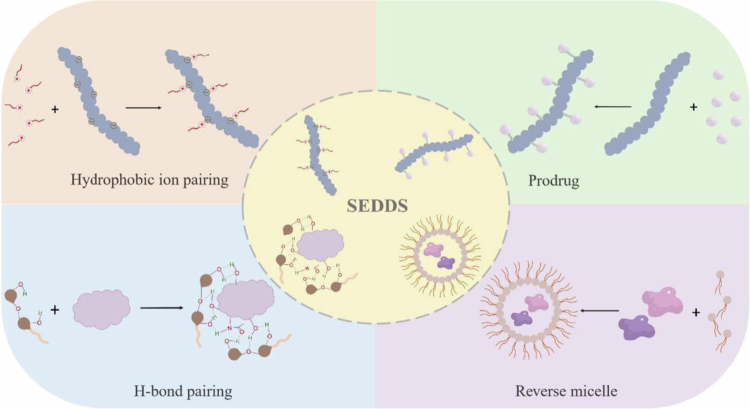
Illustration of the feasible techniques for loading therapeutic peptides and proteins in SEDDS (Drawn by the authors by use of WPS Office PPT).

SEDDSs represent a promising modality for the oral delivery of therapeutic peptides and proteins. However, prior to reaching their full potential, peptides and proteins should be well incorporated into the oily matrix of SEDDSs. The highly hydrophilic nature of peptides and proteins prevents their full dissolution in the oily matrix. Hence, peptide and protein lipidization techniques must be utilized to increase the lipophilic character of these therapeutic agents sufficiently to achieve acceptable payloads in SEDDSs. Generally, the lipidization of peptides requires non-covalent and covalent methods. Among the noncovalent methods, HIP appears to be a promising strategy for effectively improving the lipophilicity of peptides, protecting against presystemic enzymatic degradation, and enhancing intestinal membrane permeability. In addition, H-bond pairing is another typical and useful method that involves H-bond interactions. With respect to covalent methods, conjugating fatty acids to peptides *via* amidation or esterification and peptide cyclization to form lipophilic peptide prodrugs are promising strategies. However, these lipidization methods alter the lipophilicity of therapeutic peptides and, in some cases, change their structures and even cause partial or total loss of biological activity. From this viewpoint, non-covalent lipidization is preferred over covalent lipidization because a new active pharmaceutical ingredient is not created by non-covalent lipidization, and the native peptide typically regenerates *in vivo*, preserving its structure and biological activity. A reversible covalent lipidization strategy in which the native peptide regenerates from the peptide prodrug *in vivo* has also been proposed. The feasible techniques for loading peptide and protein drugs in SEDDS are illustrated in Figure 1.

### 3.1. HIP

In addition to chemical modification, HIP has proven to be the most useful technique for changing charged hydrophilic drugs into lipophilic complexes. Hydrophobic ion pairs are formed by ionic interactions between a charged hydrophilic molecule with an oppositely charged counterion possessing hydrophobic domains. Eligible therapeutic molecules for HIP must have one or more charged groups, and the charge types and charge density (charge per weight) of drug molecules are considered important factors for HIP. Generally, drug molecules with one type of charge or strong net charge easily form complexes with one counterion species. However, zwitterionic molecules are associated with the complicated problem of shielding one type of charge prior to complexation. Moreover, the inadequate charge density of the molecule probably prevents the formation of the desired hydrophobic ion pair. On the one hand, a high charge density provides comparatively high stability for the hydrophobic ion pair. On the other hand, macromolecules with a low charge density do not achieve the desired hydrophobicity in the final complex.

To construct a suitable hydrophobic ion pair, utilizing appropriate hydrophobic counterions is critical. Eligible counterions commonly contain at least one charged group and one lipophilic substructure. In the case of anionic counterions, carboxylate, phosphate, sulfonate, and sulfate have been broadly used for HIP. Carboxylates mainly consist of a variety of fatty acids (e.g. oleic acid), carboxylic acid-containing surfactants (e.g. cholic acid), and their sodium salts. In particular, sodium oleate and sodium deoxycholate are frequently selected to pair with bovine serum albumin, insulin, leuprolide, octreotide, and sCT (Table 1). The anionic carboxylic acid groups of sodium oleate and sodium deoxycholate easily bind to cationic ammonium substructures derived from the amines and amino acids of peptides and proteins. Moreover, dicarboxylic acids, such as disodium pamoate or sodium stearoyl glutamate, are also optimized in situations in which other fatty acids are ineffective (Nazir et al. 2019). Compared with carboxylates, sulfonates and sulfates are more advantageous in HIP with therapeutic peptides and proteins because of their stronger point charges. Among them, sodium docusate and sodium dodecyl sulfate are the most popular, and they have been successfully complexed with bovine serum albumin, desmopressin, leuprolide, lysozyme, and octreotide (Table 1). In addition, dextran sulfate, a polyanionic polymer, is also used for complexation with multivalent peptides or proteins. With respect to cationic counterions, only quaternary amines and alkylamines are relevant. Compared with alkylamines, quaternary ammonium groups are permanently charged and tend to bind more tightly to peptides and proteins over a broad pH range. Nonetheless, the toxicity derived from their strong cationic charges limits their use in formulations. To date, a variety of both biodegradable and less toxic cationic surfactants have been synthesized using amino acids (e.g. arginine, lysine) and fatty alcohols have been used for HIP with peptides and proteins. As a polycationic polymer, chitosan has also been used for protein complexation, although care should be taken to identify the optimal match for proteins because the distance between point charges commonly varies. In addition, divalent metal ions such as Ca^2+^, Zn^2+^, and Cu^2+^ are other alternatives for complexation with peptides. For instance, oxytocin undergoes a conformational change when coordinated with Zn^2+^, which causes the carbonyl oxygens to orient to the core of the peptide ring and renders a structured hydrophobic surface (Liu et al. 2005). Moreover, Zn^2+^ is widely used to create various water-insoluble zinc–peptide complexes, such as insulin (Ward et al. 2021), exenatide (Zhang et al. 2018), gonadorelin (Dolińska and Ryszka 2003), and hirudin (Gietz et al. 2000).

**Table 1.** Representative hydrophobic counter ions used for HIP formation with therapeutic peptides and proteins.

Counter ion	Molecular formula	Molecular weight (Da)	Structure	Peptide or protein paired
**Anions**				
Carboxy methyl polyethylene glycol	C_3_H_6_O_2_(C_2_H_4_O)n	−	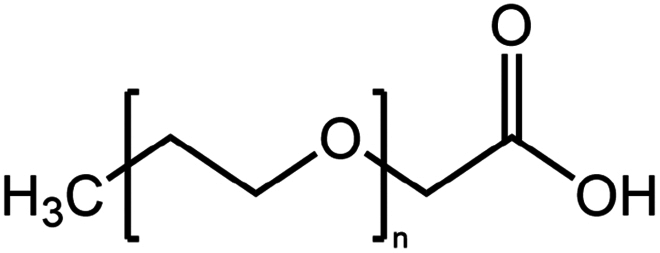	Bovine serum albumin (Fu et al. 2003) Lysozyme (Fu et al. 2003)
Cholesteryl hemisuccinate	C_31_H_50_O_4_	486.7	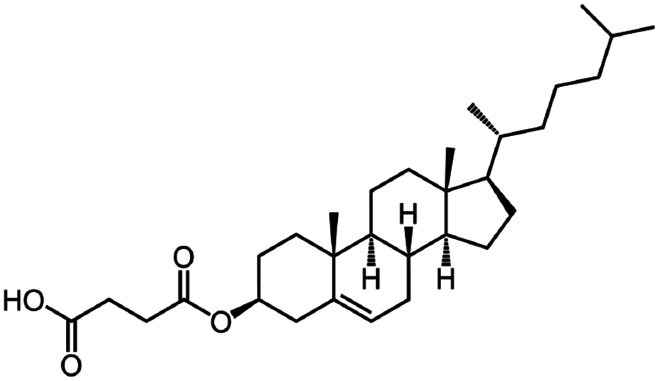	Colistin (Tang et al. 2017)
Dimyristoyl phosphatidyl glycerol (DMPG)	C_34_H_67_O_10_P	666.9	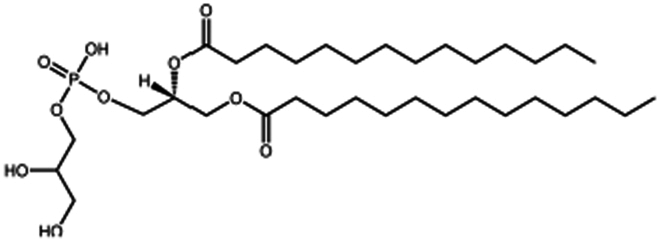	Bivalirudin (Zupančič et al. 2017) Insulin (Karamanidou et al. 2015) Salmon calcitonin (Sang Yoo and Gwan Park 2004)
Disodium pamoate (Pamoic acid)	C_23_H_16_O_6_	388.4	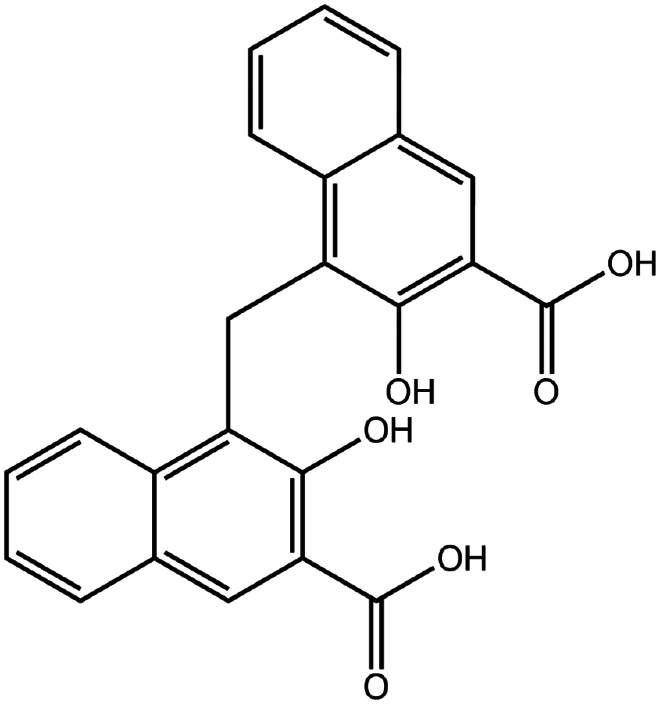	Bovine serum albumin (Nazir et al. 2019) Insulin (Nazir et al. 2019) Leuprolide (Nazir et al. 2019) Polymyxin B (Lu et al. 2018)
Dextran sulfate	(C_6_H_7_Na_3_O_14_S_3_)n	−	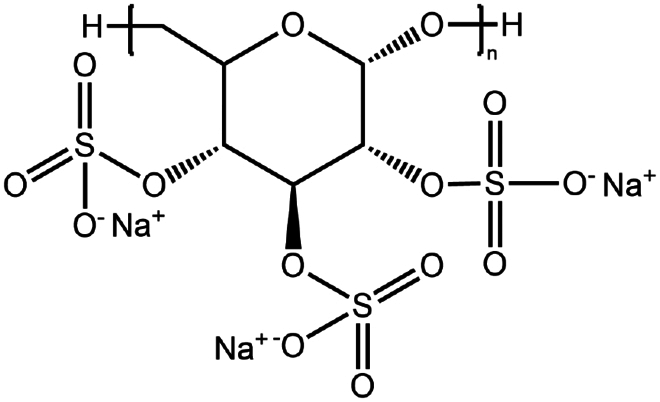	Bovine serum albumin (Gaudana et al. 2011) Dalargin (Dalwadi and Sunderland 2009) Lysozyme (Gaudana et al. 2013) Octreotide (Vaishya et al. 2015)
Hexadecylphosphate	C_16_H_35_O_4_P	322.4	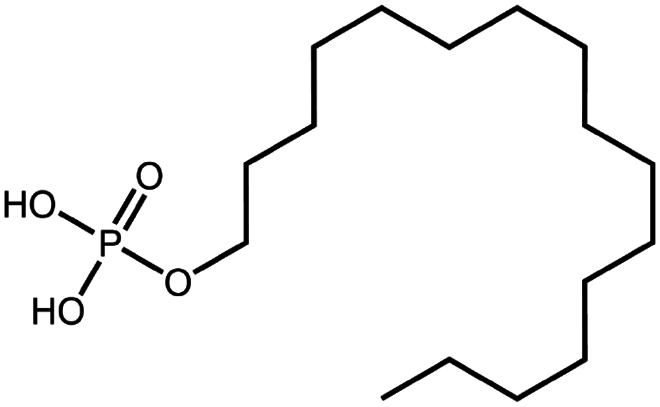	Thymopentin (Morel et al. 1996)
Linoleic acid	C_18_H_32_O_2_	280.5	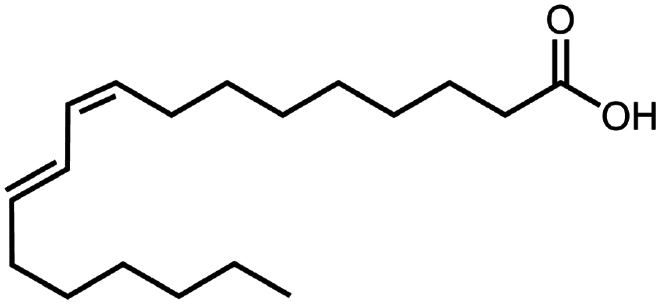	Vancomycin (Kalhapure et al. 2014)
Sodium cholate (cholic acid)	C_24_H_40_O_5_	408.6	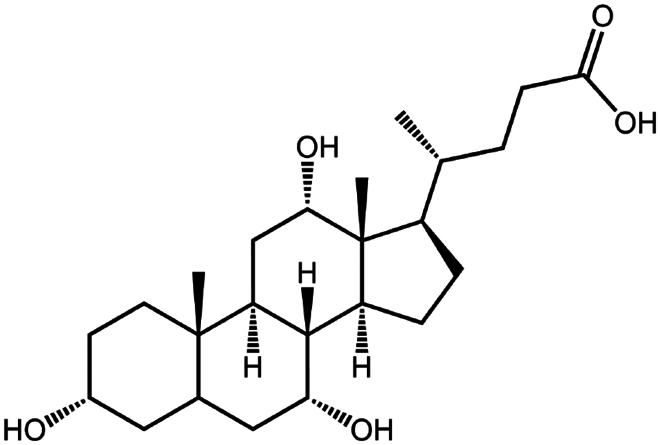	Bovine serum albumin (Fu et al. 2003) Lysozyme (Fu et al. 2003) Insulin (Liu et al. 2007)
Sodium cholesteryl sulfate	C_27_H_45_NaO_4_S	466.3	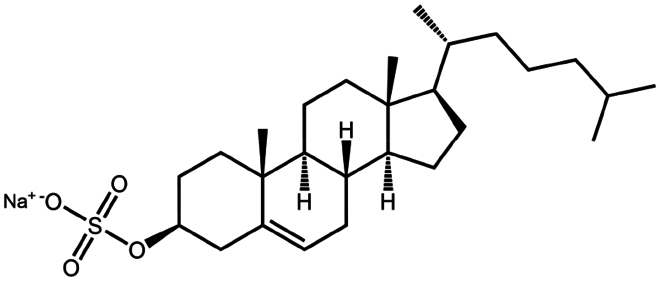	Colistin (Tang et al. 2017)
Sodium decanoate (decanoic acid)	C_10_H_20_O_2_	172.3	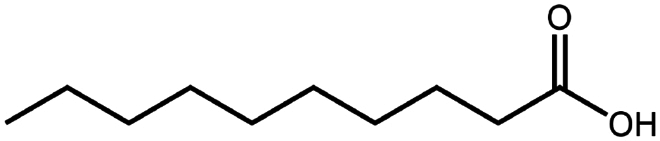	Octreotide (Bonengel et al. 2018)
Sodium deoxycholate	C_24_H_39_NaO_4_	414.6	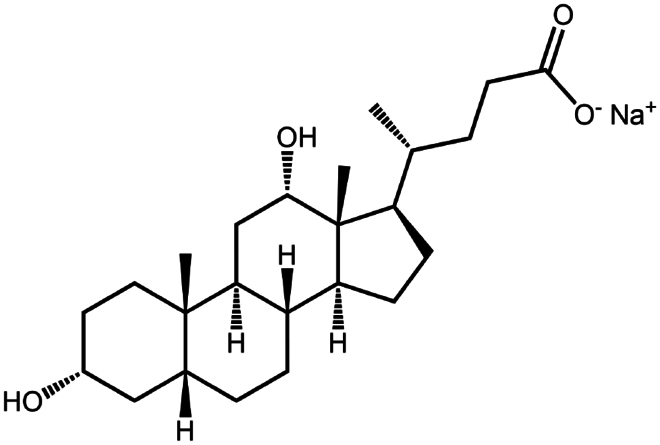	Bovine serum albumin (Nazir et al. 2019) Insulin (Nazir et al. 2019) Lanreotide (Ijaz et al. 2016) Leuprolide (Nazir et al. 2019) Octreotide (Bonengel et al. 2018) Salmon calcitonin (Sang Yoo and Gwan Park 2004)
Sodium docusate	C_20_H_37_NaO_7_S	444.6	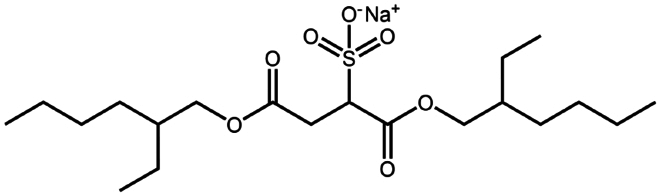	a-Chymotrypsin (Novick and Dordick 2002) Bevacizumab (Battaglia et al. 2015) Bivalirudin (Zupančič et al. 2017) Bovine serum albumin (Fu et al. 2003; Lupo et al. 2019) Desmopressin (Zupančič et al. 2016; Griesser et al. 2017; Griesser et al. 2018; Chamieh et al. 2019) Lanreotide (Ijaz et al. 2016) Leuprolide (Griesser et al. 2017; Chamieh et al. 2019; Dumont et al. 2019) Lysozyme (Fu et al. 2003) Octreotide (Bonengel et al. 2018) Trypsin (Novick and Dordick 2002) Vancomycin (Efiana et al. 2019)
Sodium dodecyl benzenesulfonate	C_18_H_29_NaO_3_S	348.5	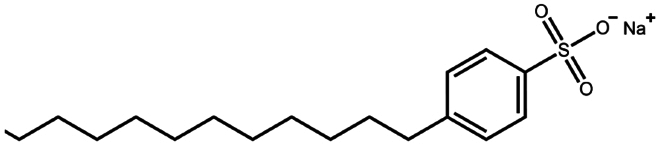	Polymyxin B (Lu et al. 2018)
Sodium dodecyl sulfate	C_12_H_25_NaO_4_S	288.4	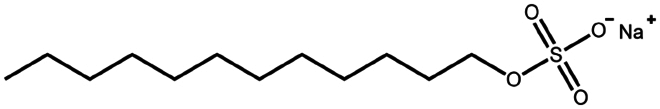	Bivalirudin (Zupančič et al. 2017) Bovine serum albumin (Fu et al. 2003) Desmopressin (Zupančič et al. 2016; Griesser et al. 2017) Insulin (Shi et al. 2008; Li et al. 2013; Griesser et al. 2017) Leuprolide (Iqbal et al. 2011; Griesser et al. 2017) Lysozyme (Yoo et al. 2001; Fu et al. 2003) Octreotide (Mahjub et al. 2015; Vaishya et al. 2015) Polymyxin B (Lu et al. 2018)
Sodium laurate	C_12_H_23_NaO_2_	222.3	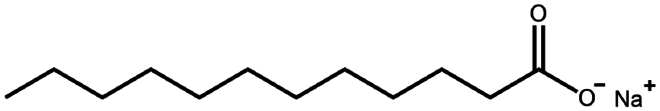	Bovine serum albumin (Nazir et al. 2019) Insulin (Nazir et al. 2019) Leuprolide (Nazir et al. 2019)
Sodium oleate (Oleic acid)	C_18_H_34_O_2_	282.5	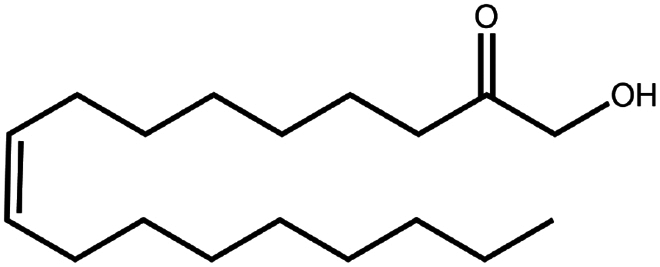	Desmopressin (Zupančič et al. 2016; Griesser et al. 2017) Insulin (Sun et al. 2015; Griesser et al. 2017) Leuprolide (Hintzen et al. 2014; Griesser et al. 2017) Lysozyme (Yoo et al. 2001) Octreotide (Bonengel et al. 2018) Polymyxin B (Lu et al. 2018) Salmon calcitonin (Sang Yoo and Gwan Park, 2004)
Sodium stearyl sulfate	C_18_H_37_NaO_4_S	372.5	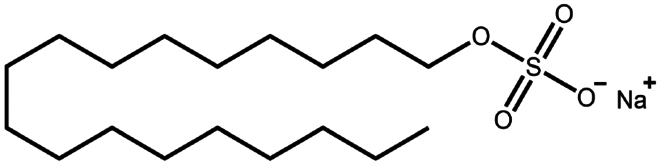	Desmopressin (Zupančič et al. 2016) Lanreotide (Ijaz et al. 2016)
Sodium stearate (stearic acid)	C_18_H_36_O_2_	306.5	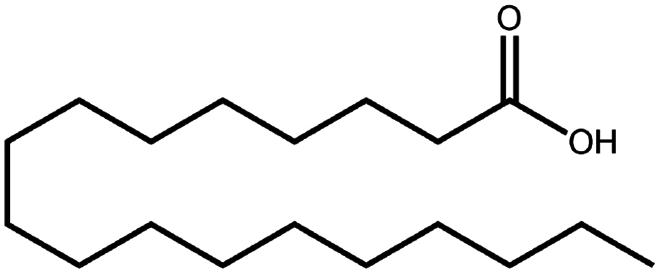	Desmopressin (Zupančič et al. 2016)
Sodium stearoyl glutamate	C_23_H_42_NNaO_5_	435.6	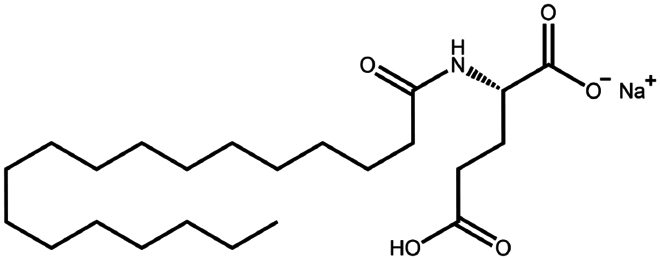	Bovine serum albumin (Nazir et al. 2019) Insulin (Nazir et al. 2019) Leuprolide (Nazir et al. 2019)
Sodium taurocholate(taurocholic acid)	C_26_H_45_NO_7_S	515.7	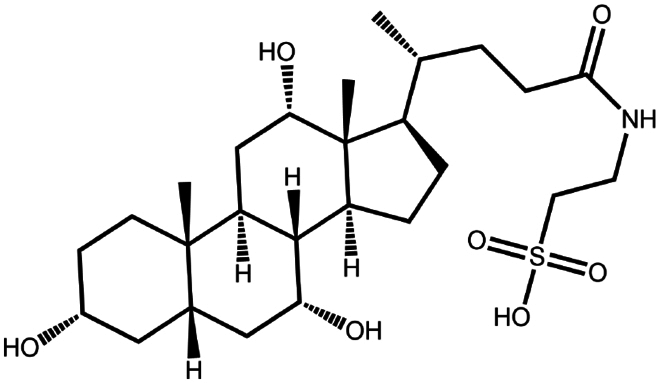	Bovine serum albumin (Fu et al. 2003) Lanreotide (Ijaz et al. 2016) Lysozyme (Fu et al. 2003)
**Amphion**				
Phosphatidylcholine	C_38_H_76_NO_8_P	−	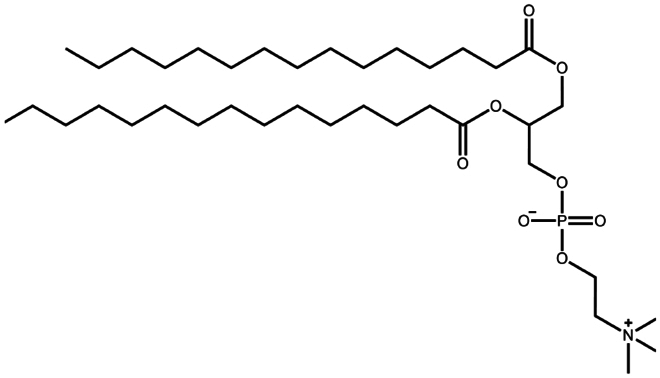	Insulin (Cui et al. 2006; Zhang et al. 2012; Li et al. 2013)
**Cations**				
Dodecylamine	C_12_H_27_N	185.3	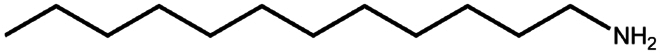	Bivalirudin (Zupančič et al. 2017) Daptomycin (Zupančič et al. 2016)
Arginine-hexadecanoyl ester (AHE)	C_22_H_46_N_4_O_2_	398.6	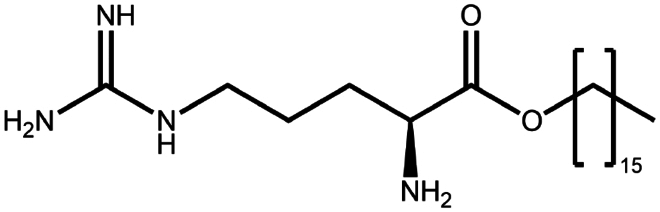	Daptomycin (Shahzadi et al. 2019)
Arginine-nonyl ester (ANE)	C_15_H_32_N_4_O_2_	300.4	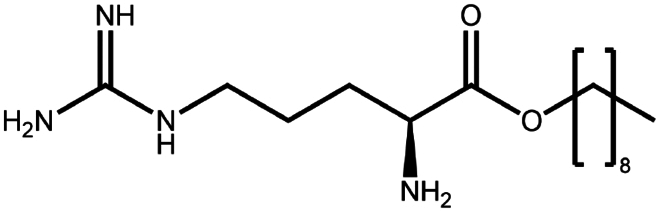	Daptomycin (Shahzadi et al. 2019)
Ethyl lauroyl arginate (ELA)	C₂₀H₄₀N₄O₃	384.5	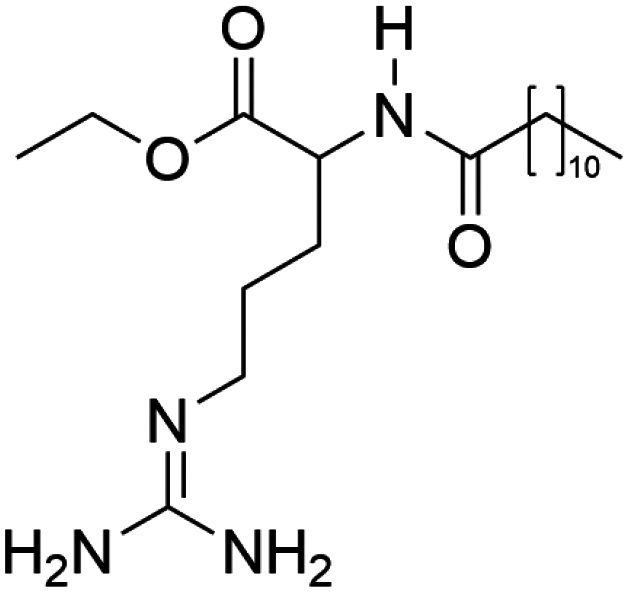	Daptomycin derivate (Truszkowska et al. 2025)
Chitosan	−	−	−	Insulin (Elsayed et al. 2011)
Distearyldimethylammonium bromide	C_38_H_80_BrN	631.0	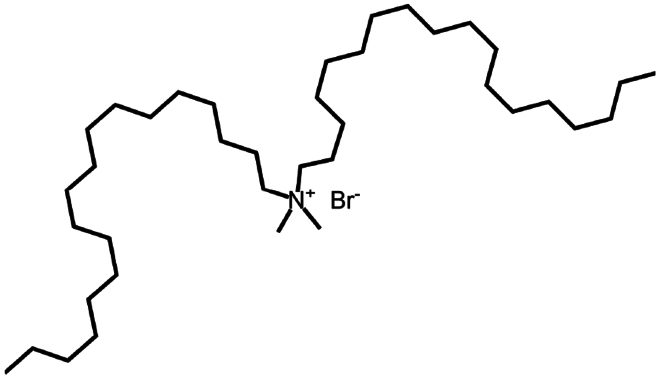	Insulin (Li et al. 2013)
Hexadecyl trimethylammonium(cetrimonium) bromide (CTAB)	C_19_H_42_BrN	364.5	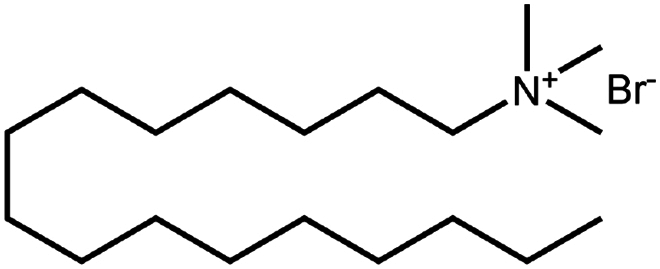	Ovalbumin (Heffernan et al. 2009)

### 3.2. H-bond pairing

In addition to HIP, H-bond pairs can form between peptides and nonionic surfactants to improve their lipophilic character. The formation of this hydrophobic complex depends on H-bonding, which is an important intermolecular or intramolecular interaction, although it is comparatively weaker than ionic bonds. H-bond pairs have great potential for the lipidization of peptides because most peptides contain at least 10-fold more H-bonding donor and acceptor substructures than ionic substructures. The first typical protein complex generated *via* H-bonding interactions was formed with tannins, which are polyphenolic compounds (Lu and Bennick 1998). This protein–tannin complex exhibited low water solubility and high stability in the GI environment (Lu and Bennick 1998). Nazir et al. reported that sucrose esters composed of sucrose as the hydrophilic head group and fatty acids as lipophilic tail groups were used to form lipophilic H-bond pairs with the peptide drug leuprolide (Nazir et al. 2020). The log *P* of leuprolide increased up to 250-fold *via* H-bond pairing with sucrose stearate. Moreover, the membrane permeation of leuprolide was obviously improved by 2-fold after hydrophobic H-bond pairing compared with that of free leuprolide. This study suggested that sucrose esters, which are widely used emulsifiers or solubilizers, can potentially increase the lipophilicity and membrane permeation of peptide drugs *via* H-bond pairing. In another study, the nonionic surfactant sorbitan oleate was utilized to form reverse micelles (RMs) for incorporating the GLP−1 analog exenatide, and the lipophilicity of exenatide was improved by RMs, permitting subsequent loading into lipid-based nanocapsules (Xu et al. 2020). Exenatide is stably anchored in micelles *via* H-bond interactions with sorbitan oleate.

### 3.3. Prodrugs

Prodrug design is a versatile chemical manipulation technique that converts a therapeutic drug into an inactive precursor, conferring improved physicochemical characteristics for drug delivery. This approach was first introduced in the late 1950s and subsequently used to produce chemical derivatives of small-molecule and macromolecular drugs. These prodrugs are designed to more readily reach therapeutic sites through transient optimization of their properties, further achieving the desired therapeutic effect. In terms of peptides and proteins, prodrugs represent a preferred modality for improving their lipophilicity beyond non-covalent methods. In reversible covalent lipidization, the native peptide regenerates from the peptide prodrug *in vivo* without risking the loss of native biological activity.

To form a lipophilic peptide prodrug, the functional groups of the peptide, such as amino, carboxyl, and hydroxyl groups at the termini or side chains, are commonly utilized for amidation or esterification with lipophilic moieties. The formed peptide esters and amides are usually sensitive to enzymes or specific chemical environments, permitting easy regeneration of the native peptides *in vivo*. As previously reported (Kahns et al. 1993), various ester prodrugs of desmopressin were developed *via* esterification of the hydroxyl group of tyrosine with different fatty acids. All of these prodrugs exhibited much higher lipophilicity than native desmopressin. Similar ester prodrugs formed *via* esterification of the hydroxyl group of tyrosine with palmitic acid were used to improve the oral absorption of leu (Fuhrmann and Leroux 2014)-enkephalin (Lalatsa et al. 2012) and a dermorphin tetrapeptide analog (Ogawa et al. 2003). Borchardt prepared several cyclic prodrugs of leu (Fuhrmann and Leroux 2014)-enkephalin using an acyloxyalkoxy linker, a phenylpropionic acid linker, and a coumarinic acid linker, which were susceptible to esterase, leading to easy release of the native peptide *in vivo* (Borchardt 1999). Compared with the parent peptide, these cyclic prodrugs displayed increased lipophilicity and membrane permeation. In addition, disulfide bonds or thiols can also be conjugated with lipid moieties. Reversible aqueous lipidization is a typical technique that has been successfully utilized for peptides and proteins containing disulfide bonds. Shen’s group synthesized a series of reversible fatty acid–desopressin conjugates *via* disulfide bonds via reversible aqueous lipidization (Wang et al. 1999, 2002). All of the conjugates were more lipophilic than desmopressin was, and the active parent drug was regenerated in the *in vitro* liver slice metabolic system (Wang et al. 2002). In another study, sCT was lipidized with *N*-palmitoyl cysteinyl 2-pyridyl disulfide using a reversible aqueous lipidization technique (Wang et al. 2003). When injected subcutaneously into mice, the AUC of the sCT prodrug was 4-fold greater than that of sCT. In addition, the AUC of the oral sCT prodrug was at least 19-fold higher than that of sCT, indicating that reversibly lipidized polypeptides led to obvious enhancement of the oral bioavailability of sCT. Moreover, the formed peptide prodrugs also protect peptides from degradation and degeneration (Ding et al. 2022).

## 4. Opportunities for using SEDDSs in the oral delivery of peptide and protein drugs

### 4.1. Maintaining intrinsic bioactivity in the GI tract

The intrinsic bioactivity of therapeutic peptides and proteins is susceptible to acidic pH, enzymes, surfactants, and exogenous or endogenous thiols in the GI environment. A prerequisite for feasible nanocarriers is that they can sufficiently protect peptide drugs before absorption. As the lipophilic characteristics of peptides and proteins are sufficiently increased by lipidization, they can be effectively dissolved in the lipophilic phase of SEDDSs. The lipophilic phase of SEDDSs effectively protects hydrophobic ion pairs, H-bond pairs, and prodrugs from exogenous or endogenous components in the GI tract because these components are typically too hydrophilic to enter the lipophilic phase. As the main surfactants in the GI tract, bile salts and fatty acids assemble on the surface of SEDDS droplets and have negligible effects on therapeutic peptides. Moreover, the oily droplets of SEDDSs commonly permit sustained release of therapeutic peptide drugs, which efficiently reduces drug loss in the GI tract.

Although most peptides and proteins are susceptible to proteolytic degradation by GI enzymes, their stability is improved to an extent in SEDDSs. In addition to the hydrophilic character of most GI enzymes, which cannot penetrate the hydrophobic core of SEDDSs, the character and composition of SEDDSs are important for resistance to hydrolysis. Leonaviciute et al. demonstrated that SEDDSs with resistance against enzymatic hydrolysis protected against leuprorelin during oral delivery (Leonaviciute et al. 2016). The amount of leuprorelin oleate in the ester-free SEDDSs was dramatically higher than that in the ester linkage-rich SEDDSs, which were susceptible to lipases in environments containing luminal enzymes. Hintzen et al. also reported that leuprorelin paired with oleate was well protected from enzymatic degradation by trypsin and *α*-chymotrypsin when embedded in SEDDSs, whereas leuprolide acetate in aqueous solution was completely metabolized by trypsin within 60 min and by *α*-chymotrypsin within 5 min (Hintzen et al. 2014). Moreover, insulin, which is susceptible to pepsin and pancreatic proteases, was used to evaluate the protective effect of the SEDDSs. A hydrophobic ion pair of insulin and dimyristoyl phosphatidylglycerol was prepared and loaded in SEDDSs to protect against enzymatic degradation (Karamanidou et al. 2015). This study revealed that >70% of insulin in solution was degraded after 180 min of incubation with trypsin, whereas 79% of insulin remained intact in the ion pair encapsulated in the SEDDSs. Similarly, only <27% of insulin embedded in SEDDSs was degraded by *α*-chymotrypsin, whereas insulin in solution was completely degraded within 60 min, demonstrating the good protective effect of SEDDSs against intestinal enzymes. It was confirmed that *α*-chymotrypsin, trypsin, and elastase were too hydrophilic to enter the oily phase of SEDDSs; thus, their enzymatic activity against leuprorelin, insulin, and desmopressin was prevented when they were paired with sodium docusate and loaded into SEDDSs (Hetényi et al. 2017). In another study, insulin paired with phosphatidylcholine was more strongly protected from *α*-chymotrypsin in a SEDDS containing medium-chain glycerides than in a SEDDS containing long-chain glycerides and was more stable in a SEDDS containing PEG−8 caprylic/capric glycerides than in a SEDDS containing PEG−40 hydrogenated castor oil (Liu et al. 2020). These results demonstrated that the protective effect of SEDDSs against GI enzymes was affected by their constituents.

Thiol/disulfide exchange reactions with exogenous or endogenous thiols can also be suppressed by incorporating bioactive compounds into SEDDSs. In Ijaz’s study, lanreotide acetate dissolved in acetate buffer was almost entirely degraded by thiol/disulfide exchange reactions with glutathione at 3 h, whereas more than 80% of lanreotide remained intact after being loaded into a SEDDS (Ijaz et al. 2016). Moreover, 80% of lanreotide was degraded within 30 min in a phosphate buffer solution containing thiol-enriched casein peptones, whereas 67% of SEDDS-encapsulated lanreotide remained after 2 h in the presence of thiol-enriched casein peptones, indicating protection against thiol/disulfide exchange reactions in oral delivery (Ijaz et al. 2016). Bernkop-Schnurch et al. developed a desmopressin docusate complex-loaded SEDDS that protected desmopressin against *α*-chymotrypsin degradation and thiol/disulfide exchange reactions with glutathione (Zupančič et al. 2016).

### 4.2. Enhancing drug absorption and transport in the GI tract

#### 4.2.1. Mucus permeation

Mucus is the main barrier limiting the oral delivery of therapeutic peptides and proteins. In addition to protecting peptides against GI enzymatic degradation, SEDDSs permit high permeation across the intestinal mucosal barrier following oral administration. The mucus-permeating properties of nanocarriers depend on their size, surface lipophilicity, surface charge, mucolytic properties, and chemical substructures. As mucus has a mesh size range of 10–200 nm, SEDDSs with a droplet size below this cutoff of 200 nm are highly desirable. A previous study revealed that the droplet size of SEDDSs significantly influences their mucus-permeating behavior. Specifically, 70.3% of the smallest droplets (12.0 nm) permeated the mucus layer, whereas only 8.3% of the largest droplets (455.5 nm) permeated the mucus layer (Friedl et al. 2013). Griesser et al. demonstrated that the permeation coefficient in mucus increases as the droplet size of SEDDSs decreases (Griesser et al. 2018).

As reported previously, SEDDSs improve the mucus permeation of peptides, probably because of the highly hydrophilic nature of the droplet surface. The addition of a glycol moiety to the compositions of SEDDSs is the most useful method for improving their hydrophilicity. The presence of PEGylated surfactants is essential for the self-emulsifying properties of SEDDSs. As surfactants or cosurfactants, these polyglycol moieties accumulate at the surface of lipid droplets and form a highly mucoinert hydrophilic surface that reduces the interactions of lipid droplets with mucus glycoproteins. Zupančič et al. used HIP to conjugate daptomycin peptide with a dodecylamine hydrochloride-loaded SEDDS consisting of an oil phase (Dermofeel MCT and Capmul MCM) and PEG-containing surfactants (Kolliphor RH40) (Zupančič et al., 2016). Compared with that of pure daptomycin, the permeation of daptomycin through pig intestinal mucus was improved by almost 2-fold in the SEDDS. Similarly, a SEDDS containing 30% Kolliphor EL and 10% propylene glycol was constructed to deliver an enoxaparin–dodecylamine complex orally, and high mucus diffusion and increased oral bioavailability of enoxaparin were observed (Zupančič et al. 2016). PEG-coated SEDDSs are widely exploited to increase the intestinal mucus permeation of therapeutic peptides for diabetes. A hydrophobic ion pair of insulin and dimyristoyl phosphatidylglycerol was prepared and loaded in a SEDDS with the PEG emulsifier Kolliphor EL and coemulsifier Transcutol *P*, and 40% of the construct permeated through the purified porcine intestinal mucus after 6 h (Karamanidou et al. 2015). In another study, exenatide paired with sodium docusate was incorporated into a SEDDS consisting of 35% Kolliphor EL and 10% propylene glycol as surfactants (Menzel et al. 2018). The mucus permeation of exenatide in this SEDDS was enhanced by 2.7-fold compared with that of subcutaneously administered exenatide, and its oral bioavailability was increased by 14.6%. Furthermore, Abdulkarim et al. reported an octreotide-loaded SEDDS containing the PEG emulsifier Brij O10, and extremely high permeation across the intestinal mucus and an obvious increase in the oral bioavailability of octreotide were demonstrated (Bonengel et al. 2018). Additionally, the mucus diffusion of the SEDDS droplets increases with increasing density of the PEG coating until a plateau is reached. Recently, Sandmeier et al. investigated the ability of surface decorations of SEDDSs to improve mucus and intestinal membrane permeability through the selection of different surfactant heads, such as polyglycerol (PG), phosphatidyl choline (PC) and PEG-ether (Sandmeier et al. 2025). The surface hydrophobicity of the SEDDSs was in the order of PEG-based SEDDSs >PC-based SEDDS > and PG-based SEDDSs, which were inversely correlated with the water binding and H-bonding capabilities of the surfaces. As a result, the PEG-based SEDDS achieved the highest mucus permeation and cellular internalization, indicating that the high surface hydrophobicity tuned by PEG was superior for the ability of the SEDDS to cross oral absorptive barriers (Sandmeier et al. 2025). These studies suggest that both the surface hydrophilicity and lipophilicity of SEDDSs are important factors that increase their performance in terms of GI absorption (Lei et al. 2017). In other words, optimizing the most suitable surface modifications of SEDDSs by achieving ‘hydrophilicity/hydrophobicity balance’, which requires good design and sufficient screening, is effective.

**Figure 2.**
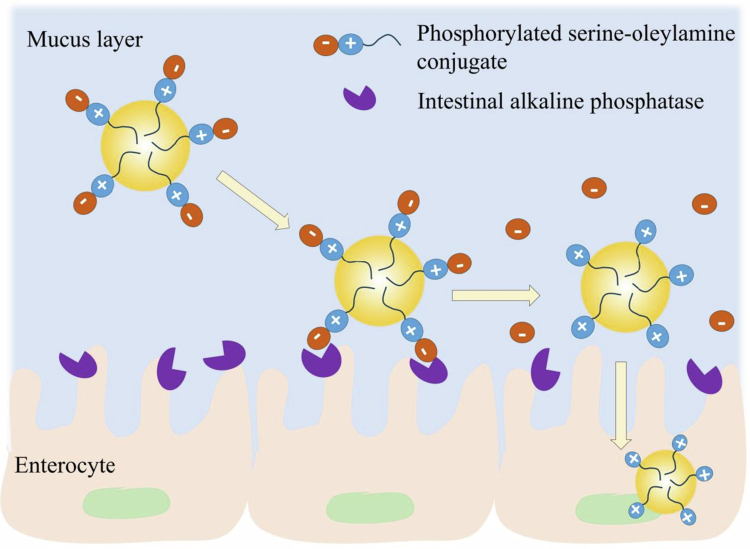
Illustration of a zeta potential-changing SEDDS modified by phosphorylated serine–oleylamine that is cleaved off by intestinal alkaline phosphatase on the intestinal epithelium brush border with enhanced mucus permeation and cellular uptake (Drawn by the authors by use of WPS Office PPT).

The surface charge is another primary factor influencing the mucus-permeating properties of SEDDSs. SEDDSs with different zeta potentials exhibited varying mucus-permeating behaviors. A neutral or negative charge is advantageous for nanocarriers to achieve high mucus permeability via the avoidance of ionic interactions with the negatively charged mucins in the mucus gel layer. Similarly, Griesser et al. demonstrated that a negatively charged SEDDS exhibited a higher permeation rate than a positively charged SEDDS did (Griesser et al. 2018). However, after passing through the intestinal mucus, the negative charge of the SEDDS results in poor interactions with negatively charged epithelial cells, thereby limiting their fusion with the cell membrane or endocytosis before absorption. To solve this problem, a zeta potential-changing strategy capable of reversing the charge from negative to positive upon reaching the cell membrane has been employed to achieve both high mucus permeation and high cell permeation (Arshad et al. 2024). Bernkop-Schnürch and colleagues first developed a zeta potential-changing SEDDS using 1,2-dipalmitoyl-sn-glycero−3-phosphatidic acid sodium (PA), a substrate of intestinal alkaline phosphatase (AP), which is expressed on the intestinal epithelium brush border (Bonengel et al. 2015; Suchaoin et al. 2016). The negatively charged surface of the SEDDS, which favored efficient mucus permeation, became positive after rapid enzymatic cleavage of the anionic phosphate substructure of PA by intestinal APs, leading to increased membrane permeability (Suchaoin et al. 2016). Further work was conducted to develop various phosphorylated surfactants that could be incorporated into SEDDSs to change the surface charge through the same mechanism of AP cleavage. Phosphorylated polysaccharides were formulated in a SEDDS by Griesser, and mucus diffusion was improved by 3-fold compared with that in a PA-loaded SEDDS because of the enzyme-induced zeta potential reversal (Griesser et al. 2017). Moreover, Bernkop-Schnürch’s group synthesized a series of phosphorylated conjugates containing both positively and negatively charged groups in one conjugate, such as phosphorylated tyrosine–octadecylamine (Salimi et al. 2018), phosphorylated serine–oleylamine (Nazir et al. 2019), and *N*,N′-bis(polyoxyethylene)oleylamine bisphosphate (Wolf et al. 2020). After loading into the SEDDSs, these conjugates provided a flip–flop mechanism that was controlled by phosphate cleavage, specifically enhancing mucus permeation *via* a negative zeta potential because of the phosphate substructures and subsequently facilitating cellular uptake through a change in zeta potential after the phosphate substructures were cleaved by AP, as illustrated in Figure 2. As previously reported (Nazir et al. 2019), compared with control SEDDSs, phosphorylated serine–oleylamine-loaded SEDDSs exhibited improved mucus permeation and almost 2-fold greater cellular uptake after phosphate cleavage. Surely, the success of the zeta potential-changing strategy depends on both the chemical structure of the phosphorylated excipient and the SEDDS composition, especially the effect of the PEG corona of the SEDDS on the activation of enzymatic stimuli (Akkuş-Dağdeviren et al. 2021).

The mucolytic strategy is a rational approach to achieve improved mucus permeation by SEDDSs. Generally, a mucolytic agent is loaded into the SEDDS to decrease the viscosity of the top of the mucus layer and reduce the resistance to the penetration of the droplets through the mucus without disrupting the entire mucus barrier. Leichner et al. created a SEDDS loaded with the mucolytic protease papain and evaluated its permeation of porcine intestinal mucus. Compared with the papain-free SEDDS, the SEDDS packaged with a papain–deoxycholate ion pair exhibited a mucolytic effect and 3-fold greater mucus diffusion (Leichner et al. 2017). In another study, Efiana et al. effectively incorporated papain into a SEDDS *via* lipidization with palmitoyl chloride, and the permeation of the SEDDS through porcine intestinal mucus was enhanced by 4.6- and 2-fold, as evaluated by the Transwell diffusion assay and rotating tube method, respectively (Efiana et al. 2018). Moreover, Shahzadi et al. prepared a trypsin-decorated SEDDS and demonstrated that mucus permeation was improved by 1.6−2.6-fold in comparison with that of the blank SEDDS (Shahzadi et al. 2018). Mucolytic proteases can disrupt the peptide bonds of mucins, leading to reversible breakage of mucosal substructures and reducing the viscosity of mucus (Mahmood et al. 2022). In addition to mucolytic enzymes, thiol-bearing agents are also used to develop mucus-permeating mucolytic SEDDSs by breaking the disulfide bonds of the mucus network. Rohrer et al. incorporated thiobutylamidine–dodecylamine and thioglycolic acid–octylamine into the oil phase to obtain thiolated SEDDSs, which exhibited mucolytic activity and an effective diffusion coefficient of 0.871 × 10^−9^ ± 0.122 × 10^−9^ cm^2^ s^−1^, which was 60-fold greater than that of the unthiolated SEDDS (Rohrer et al. 2016).

#### 4.2.2. Cell permeation

**Figure 3.**
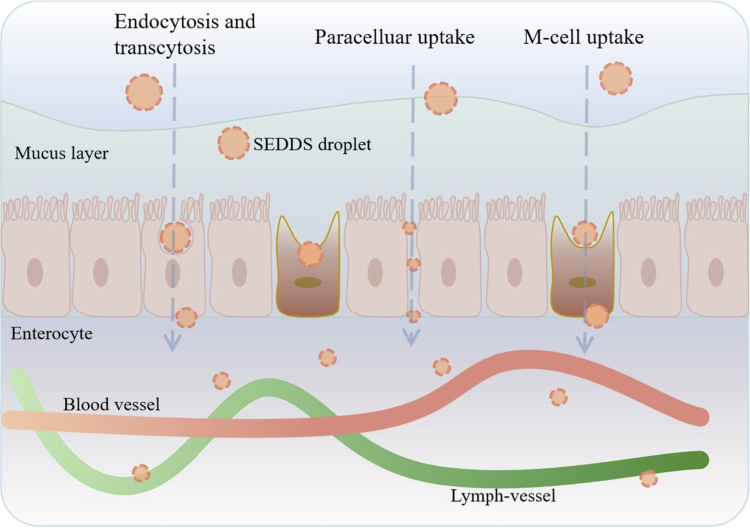
Illustration of different pathways of the SEDDS droplets across the intestinal epithelium (Drawn by the authors by use of WPS Office PPT).

The transport of therapeutic peptides and proteins across the GI mucosa is a complicated process that limits the oral delivery of these drugs. Lipid-based nanocarriers increase the ability of peptides to cross the intestinal cell monolayer through several mechanisms, including endocytosis and transcytosis, the paracellular route, and M cell-mediated transport, as illustrated in Figure 3. As lipid nanocarriers, SEDDSs tend to facilitate paracellular uptake, probably because of their small droplet size and presence of surfactants. Bunchongprasert et al. observed enhanced permeation of SEDDS droplets with a mean size of 30 nm through an MDCK cell monolayer by opening tight junctions, but this was not observed for 250-nm droplets (Bunchongprasert and Shao 2019). Bonengel et al. reported that SEDDSs improved transport through the porcine intestinal mucosa by opening tight junctions, possibly because surfactants with a high HLB, such as Kolliphor EL, Tween 80, or Brij O10, can loosen tight junctions (Bonengel et al. 2018). Consequently, octreotide–deoxycholate-loaded and octreotide–docusate-loaded SEDDSs presented 17.9- and 4.2-fold higher relative bioavailabilities than the control, respectively, demonstrating the excellent permeation-enhancing effect of the SEDDSs (Bonengel et al. 2018). In another study, an insulin–phospholipid complex-loaded SEDDS was developed, and the transport of insulin across a Caco−2 cell monolayer was obviously enhanced by 9.3-fold compared with that of insulin in solution because of the opening of cellular tight junctions (Li et al. 2014). Finally, this SEDDS increased the relative bioavailability of insulin by 2.7-fold, and its glucose-reducing effect was increased by 3.4-fold (Li et al. 2014).

Moreover, the lipid droplets derived from SEDDSs can be internalized by endocytosis and transcytosis. Alqahtani et al. demonstrated that clathrin-mediated endocytosis was involved in the cellular uptake of SEDDSs in an inhibition study using a clathrin-dependent endocytic inhibitor (Alqahtani et al. 2013). Mahmood et al. designed an HIV−1 Tat–oleoyl conjugate-decorated SEDDS that increased the cellular uptake of lipid droplets by 2.3- and 2.6-fold after 2 and 4 h of incubation with Caco−2 cells, respectively (Mahmood et al. 2016). The subsequent endocytosis inhibition study indicated that clathrin-mediated endocytosis and caveolae-mediated endocytosis were the major pathways for cellular internalization. As previously discussed, SEDDSs with surface charge-changing properties could achieve improved mucus permeation and enhanced cellular permeation. In addition, intestinal epithelial cell membrane receptor- or transporter-targeting components, as reviewed previously (Bert et al. 2012; Xingwang and Wei 2014; Feiyang and You Han 2020), can be selected to decorate the surface of SEDDS droplets to further enhance the cell permeation of therapeutic peptides and proteins. For instance, bile acids can be used not only as hydrophobic counterions to form ion pairs with peptides or proteins for drug loading but also to decorate nanocarriers to target sodium-dependent bile acid transporters on the intestinal epithelium for enhanced cell permeation. As reported previously (Weiwei et al. 2018; Feiyang and You Han 2020), bile acid-containing oral nanocarriers have exhibited high transport efficacy for ASBT-mediated absorption and lymphatic delivery.

#### 4.2.3. Lymphatic targeted delivery

The lymphatic pathway is an alternative pathway for oral drug delivery. Compared with portal transport, lymphatic transport directs drugs entering the lymphatic system directly to the systemic circulation, avoiding hepatic first-time metabolism. As previously acknowledged, the chylomicrons synthesized in enterocytes and M cells in Peyer’s patches are the two main targets for achieving efficient lymphatic transport. Lipid-based carriers, especially those containing long-chain fatty acids, have been optimized to increase the lymphatic transport of lipophilic drugs through associations with chylomicrons in enterocytes. On this basis, it was suggested that the lipidization of peptides and proteins, such as HIP or fatty acid prodrug design, could facilitate their association with chylomicrons and thereby enhance the lymphatic transport of these hydrophilic macromolecule drugs (Niu et al. 2016). Moreover, peptide-loaded lipid nanocarriers that permeate the intestinal epithelium intact can be absorbed *via* the lymphatic pathway once they enter the lymphatic system through the leaky parts of the lymphatic vessels (Trevaskis et al. 2015). Although the pathways and extent of transport of peptides or lipid nanocarriers across the intestinal epithelial barrier have not been clearly identified, antigen sampling and presentation via M cells, the paracellular pathway, or the transcellular pathway might play important roles in the lymphatic transport of macromolecule drugs and nanocarriers.

Compared with other nanocarriers, SEDDSs enriched with various lipid components tend to improve the oral delivery of peptides and proteins by facilitating their lymphatic transport. Recently, it was reported that both lipid components and certain surfactants in SEDDSs promote lymphatic transport (Lind et al. 2008). Sha et al. designed a self-microemulsifying drug delivery system (SMEDDS) to increase the oral absorption of probucol, a model drug of choice for the study of lymphatic transport (Sha et al. 2012). Compared with a probucol suspension, the SMEDDS dramatically increased the oral bioavailability of probucol by 10.22-fold, which was probably attributable to increased lymphatic transport (Sha et al. 2012). In another study, a morin–phospholipid complex-based self-nanoemulsifying drug delivery system (SNEDDS) was developed to evaluate the contribution of lymphatic transport to the total oral absorption of morin (Zhang et al. 2015). The lymphatic transport of the morin–phospholipid complex was not significant unless it was entrapped in the SNEDDS. As visualized by fluorescence imaging in this work, it was suggested that both the chylomicrons in enterocytes and the M cell pathway were involved in the lymph transport of this SNEDDS. Moreover, the SNEDDS could be internalized in an intact form into enterocytes in a cholesterol-dependent manner via clathrin-mediated endocytosis and micropinocytosis or *via* the uptake of M cells in Peyer’s patches, resulting in enhanced lymphatic transport. Furthermore, the anticancer peptide LyP−1 was loaded into a solid SEDDS for lymphatic targeted delivery to treat breast cancer (Timur and Gürsoy 2020). The excipients used in the solid-SEDDS enhanced the lymphatic transport of LyP−1 and improved its anticancer activity in MDA-MB−231 cells.

### 4.3. Engineering potential SEDDSs to overcome the intestinal mucosal barrier

**Figure 4.**
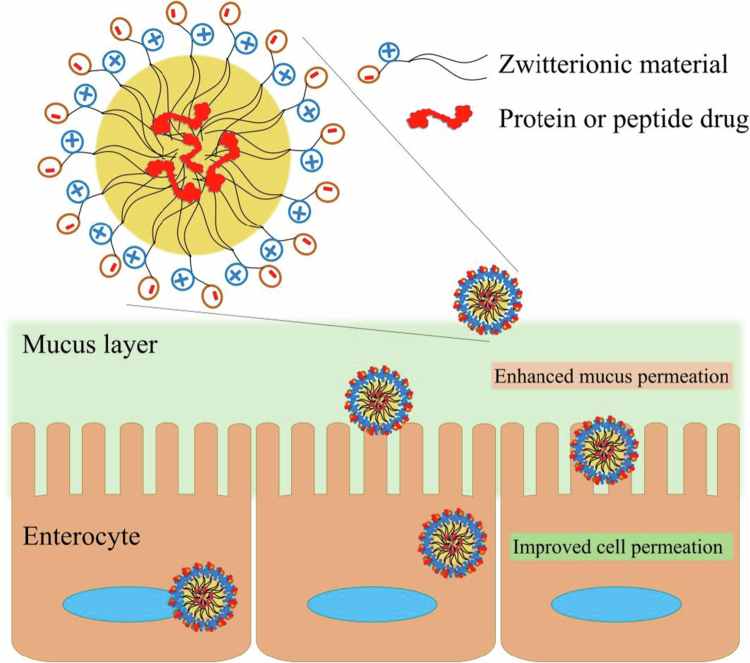
Illustration of a zwitterionic material decorated nanocarrer possessing a virus-mimetic surface with enhanced mucus and cell permeation (Drawn by the authors by use of WPS Office PPT).

As an alternative to surface PEG coating, zeta potential changes, and other strategies, zwitterionic materials might be utilized to construct high-permeating SEDDS for oral peptide delivery. Zwitterions are electrically neutral compounds composed of a cation and anion, and they commonly resist interactions with proteins in complex environments. Zwitterions can be anchored to the carrier surface to form a zwitterionic surface that shares some features with mucus-permeating viruses. The strong surface hydration might explain the high resistance of the zwitterionic surface to nonspecific protein adsorption. In detail, a zwitterion unit was able to bind approximately eight water molecules, whereas a PEG unit could bind only one water molecule (Wu et al. 2012). This hydration behavior of the virus-mimicking zwitterionic surface led to a high mucoinsertion property to avoid interactions with the intestinal mucus. Phospholipids, as the most common zwitterionic lipids, have been widely used to create zwitterionic surfaces of lipid nanocarriers. Shan et al. developed zwitterion-based self-assembled nanoparticle (NP) for the oral delivery of insulin using dilauroyl phosphatidylcholine (DLPC) (Shan et al. 2016). The DLPC coating gave the NPs a neutral and hydrophilic zwitterionic surface and obviously enhanced the permeation of the NPs through mucus, similar to F127-coated NPs, which are typical mucus-permeating particles. Moreover, the DLPC NPs also exhibited excellent affinity for intestinal epithelial cells and 4.5-fold higher cellular uptake than F127 NPs. Ultimately, the insulin-loaded DLPC NPs exerted a greater hypoglycemic effect and 6.9-fold greater oral bioavailability than free insulin, suggesting the potential for zwitterion surface decoration. In addition to phospholipids (Zhang et al. 2024), zwitterionic betaine polymers, such as poly(phosphobetaine) (Rao et al. 2021), poly(sulfobetaine) (Gao et al. 2021), and poly(carboxybetaine) (PCB) (Han et al. 2020; Li et al. 2021), are promising zwitterions for achieving both improved mucus permeation and transepithelial absorption, as illustrated in Figure 4. Han reported a zwitterionic micelle possessing a virus-mimetic zwitterionic surface using DSPE-PCB for the oral delivery of insulin (Han et al. 2020). This carrier diffuses much more rapidly (12-fold) in mucus than mucus-penetrating PEG-based NPs do and drastically enhances the intestinal transport of insulin *via* transporter-mediated epithelial absorption, simultaneously overcoming both the mucus and epithelial barriers. Compared with the common mucoinert polymer PEG, zwitterions are more likely to be chosen for mucus-penetrating nanocarriers. Haddadzadegan reported a comparative analysis of zwitterionic surfactant-based and PEG-based SEDDSs for the oral delivery of insulin glargine (Soheil et al. 2024). Compared with the PEG-based SEDDS, the zwitterionic surfactant-based SEDDS loaded with insulin glargine HIP presented a 1.85-fold greater oral bioavailability. More importantly, zwitterions exhibit stronger hydration through electrostatic interactions than does PEG through hydrogen bonding, resulting in high protein adsorption resistance and mucus inertness. Additionally, zwitterions possess unique advantages in terms of membrane affinity compared with PEG coatings. Thus, zwitterion functionalization could be a potential strategy for SEDDS development to overcome the intestinal mucosal barrier.

As an alternative method to the lipidization of peptide drugs (e.g. HIP) for loading into SEDDSs, RMs can potentially be used to incorporate hydrophilic drugs into the oil droplets of SEDDSs. Jörgensen et al. constructed dry reverse micelle (DRM)-containing SEDDSs for the oral delivery of the therapeutic peptide polymyxin B (Jörgensen et al. 2023). In this study, anionic sodium docusate (AOT), cationic dimethyl dioctadecyl ammonium bromide (DODAB), amphoteric soy lecithin (SL), and nonionic polysorbate 85 (P85) were employed to form RMs in the oily phase, and promising DRMs for loading polymyxin B were formed by 0.25% P85, 5% AOT, 10% DODAB, and 10% SL. P85-SEDDS featured the lowest payload of 0.51%, whereas SL-SEDDS achieved the highest payload of 1.99%. Moreover, the polymyxin B payloads of DRM-containing SEDDSs were obviously higher than those obtained from hydrophobic ion pairs (up to 0.29%) because the higher lipophilic character of polymyxin B-loaded DRMs resulted in higher solubility in the oily phase of SEDDSs. Specifically, for HIP, only eight hydrophobic anionic counterions can be bound to the amine substructures of polymyxin B, whereas for DRMs, all the hydrophilic substructures on the peptide are shielded by surfactant molecules. In addition, DODAB-SEDDS most efficiently improved cellular uptake, reaching almost 100% within 4 h and being 10–40-fold higher than that of the other SEDDSs, which was attributed to the cationic surface charge of DODAB-SEDDS and its affinity for the negatively charged cell membrane, facilitating its uptake by Caco−2 cells. Promising oral antimicrobial activity was obtained for all polymyxin B-loaded DRM-containing SEDDSs, revealing a potential strategy for oral peptide delivery. In another study, an RM-loaded SEDDS was prepared to enhance the oral delivery of exenatide for type 2 diabetes treatment (Lu et al. 2023). Phospholipids were employed to form RMs in the oil phase, with the hydrophobic part orienting toward the oil and the hydrophilic part forming a polar core for exenatide loading. Then, Kolliphor RH40 and caprylocaproyl macrogol−8 glycerides were added as surfactants to the oily solution to obtain RM-SEDDSs. Consequently, the RM-SEDDSs exhibited more efficient cellular uptake and transmembrane transport of exenatide into intestinal epithelial cells than SEDDSs. The RM-SEDDSs enhanced drug cell membrane permeability by disrupting the tight junction protein ZO−1 and improving cell membrane fluidity. Finally, a pharmacodynamic study demonstrated that the RM-SEDDSs achieved better control of blood glucose levels, improved pancreatic *β* cell function, and reduced insulin resistance and hyperlipemia complications in rats with type 2 diabetes. A study also provided a comparative analysis of HIP- and DRM-containing SEDDSs in improving the oral absorption of exenatide, and they demonstrated similar potential in terms of oral bioavailability (Schmidt et al. 2025). Thus, these RM-containing SEDDSs are expected to achieve efficient oral delivery of various therapeutic peptides.

## 5. Challenges and perspectives

**Figure 5.**
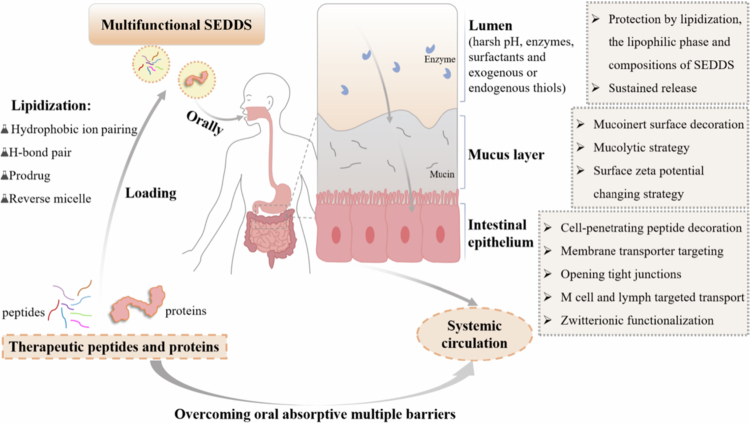
Utilization of SEDDS in oral delivery of therapeutic peptides and proteins (Drawn by the authors by use of WPS Office PPT).

The potential of SEDDSs in the oral delivery of therapeutic peptides and proteins is summarized according to current knowledge. (1) High drug loading: The developed lipidization methods provide sufficient opportunities to achieve acceptable payloads for hydrophilic peptides and proteins in SEDDSs. (2) Maintaining intrinsic bioactivity: Peptides and proteins can be protected from proteases and thiol/disulfide exchange reactions through efficient lipidization methods combined with optimized SEDDS compositions. (3) Enhancing drug absorption and transport: High mucus and membrane permeability could be acquired through multifunctional SEDDSs based on the optimized functional excipients (Figure 5). Hence, many *in vivo* studies have demonstrated high oral bioavailability and desirable therapeutic effects for peptide and protein drugs loaded into multifunctional SEDDSs (Table 2). In addition to the antidiabetic and hormonal peptides summarized in Table 2, SEDDSs also have potential in the oral delivery of immunotherapeutic or antimicrobial peptides. Lipid-based carriers have advantages in improving the oral absorption of programmed death−1/programmed cell death-ligand 1 blocking peptides (Li et al. 2021; Yang et al. 2024; Chenqi et al. 2025) and antimicrobial peptides (Gao et al. 2025). The SEDDSs will subsequently exhibit broad applicability in the oral delivery of therapeutic peptides and proteins. However, it remains difficult to identify the optimal excipients and combination forms to obtain multifunctional SEDDSs that possess all the desired properties. First, the characteristics of the selected excipients and the functionalities introduced to the SEDDS should be thoroughly clarified. Efforts should be devoted to identifying feasible and efficient combination forms of excipients and therapeutic peptides for SEDDSs. *In vitro* and *in vivo* evaluation methods, including newly established imaging techniques, are commonly employed to identify the effects of excipients in the optimized combination. Moreover, artificial intelligence aids will assist in the development of SEDDSs (Tan et al. 2023). Second, only a few details about the interactions of certain excipients with the absorptive membrane and the *in vivo* fate of SEDDSs are known. The utilization of more advanced analytical tools would contribute to a deeper understanding of the fate of excipients and SEDDSs during absorption and transport processes, further facilitating the successful development of multifunctional SEDDSs.

**Table 2.** Enhancing oral absorption of therapeutic peptides and proteins by utilizing multifunctional SEDDS *in vivo.*

Therapeutic peptide/protein	Lipidization method	Favored property	*In vivo* outcome	Reference
Insulin	Complex with phospholipid	Improved cell permeation	2.7-fold improvement in oral bioavailability in rats	Li et al. (2014)
Insulin glargine	HIP with sodium octadecyl sulfate	−	7.7-fold improvement in oral bioavailability in rats	Claus et al. (2023)
Leuprolide	HIP with sodium oleate	Stability against enzymatic metabolism	17.2-fold improvement in oral bioavailability in rats	Hintzen et al. (2014)
Exenatide	HIP with sodium docusate	Improved mucus permeation	A relative bioavailability (versus s.c.) of 14.62% in rats	Menzel et al. (2018)
Exenatide	HIP with tetraheptylammonium bromide or sodium docusate	Improved memberane permeation	A relative bioavailability (versus s.c) of 27.96% or 16.29% in rats	Ismail et al. (2020)
Exenatide	HIP with toctadecyl sulfate or sodium docusate	Improved memberane permeation	A relative bioavailability (versus s.c) of 19.6% or 15.2% in rats	Phan et al. (2020)
Octreotide	HIP with deoxycholate or docusate	Improved mucus permeation	17.9 fold or 4.2 fold improvement in oral bioavailability in pigs	Bonengel et al. (2018)
Salmon calcitonin	HIP with sulfosuccinate	−	A higher relative pharmacological activity (13.8%) in rats	Wibel et al. (2023)
Exenatide	Reverse micelles	Improved cellular uptake and transmembrane transport	Better control of blood glucose in type 2 diabetic rats	Lu et al. (2023)
Insulin glargine	HIP with bis(isotridecyl)sulfosuccinate	−	An absolute bioavailability of 2.13% in rats	Soheil et al. (2024)
Horseradish peroxidase	Dry or wet reverse micelles	Enhanced membrane permeability	An absolute bioavailability of 11.2% or 7.9% in rats	Eczacioglu et al. (2025)

## 6. Conclusion

During the past several decades, SEDDSs have displayed great potential as effective systems for the oral delivery of therapeutic peptides and proteins. As reviewed in this study, all of the main barriers encountered in the oral delivery of peptide and protein drugs, including acidic pH, enzymes, sulfhydryl barriers, and mucus and epithelium barriers, could be appropriately addressed with well-designed SEDDSs. Peptides and proteins can be protected from proteases and thiol/disulfide exchange reactions through efficient lipidization methods combined with optimized SEDDS compositions. Although mucoinert agents could obviously improve the mucus-permeating properties of SEDDS droplets, the mucoinert surface of the droplets limits interactions with the absorption membrane. Novel multifunctional auxiliary agents, such as charge-changing compounds and zwitterions, confer high mucus and cellular membrane permeation to SEDDSs. RM-containing SEDDSs represent efficient modalities for the efficient oral delivery of various therapeutic peptides. Along with the introduction of more novel multifunctional auxiliary agents and complicated structures to new-generation SEDDSs, more work is needed to increase our knowledge of the fate of excipients and nanocarriers during absorption and transport. This knowledge will contribute to the sophisticated design of more efficient SEDDSs for the oral delivery of therapeutic peptides and proteins in the future.

## Data Availability

The data that support the findings of this study are available from the corresponding author upon reasonable request.
